# The Independent and Joint Effects of Different Childhood Abuse Types on Subjective Prospective and Retrospective Memory Impairment During Pregnancy

**DOI:** 10.3389/fpsyg.2021.753008

**Published:** 2021-11-19

**Authors:** Xuan Zhang, Liuliu Wu, Juan Wang, Fangxiang Mao, Jiwei Sun, Danfeng Cao, Fenglin Cao

**Affiliations:** ^1^School of Nursing and Rehabilitation, Shandong University, Jinan, China; ^2^Shandong Province Qianfoshan Hospital, Jinan, China

**Keywords:** childhood abuse, pregnancy, memory impairment, emotional abuse, physical abuse, sexual abuse

## Abstract

**Background and Objective:** Childhood abuse is considered a risk factor in various health outcomes during pregnancy. However, no study has explored the relationship between childhood abuse and memory impairment during pregnancy. This study is the first to explore the relationship between childhood abuse and subjective memory impairment.

**Participants, Setting, and Methods:** A total of 1,825 pregnant women were recruited from a comprehensive hospital in Shandong province, China, and completed a questionnaire survey. Multivariable linear regression analysis was used to explore the relationship between childhood abuse and subjective prospective and retrospective memory.

**Results:** Pregnant women with high total childhood abuse scores had high prospective and retrospective memory impairment. Among pregnant women reporting only emotional abuse, only physical abuse, or only sexual abuse, women reporting only emotional abuse were found to have high prospective and retrospective memory impairment. Women with all three childhood abuse types also had high prospective and retrospective memory impairment.

**Conclusion:** Women who experienced childhood abuse, especially childhood emotional abuse, had high subjective memory impairment during pregnancy. It is important to ask pregnant women about their experiences of childhood abuse, especially emotional abuse, during early prenatal care, as such abuse is likely to have negative effects on memory during pregnancy.

## Introduction

Childhood abuse, including physical abuse (PA), emotional abuse (EA), and sexual abuse (SA), has become a public concern in China. It has been estimated that 26.6% of children suffer from PA, 19.6% suffer from EA, and 8.7% suffer from SA in China (Fang et al., 2015). CA is one of the strong social and environmental predictors for many of the leading causes of death, disease, and disability during all stages of life (Viola et al., 2016). Among these, pregnancy is a vulnerable stage, during which CA might play an important role. CA has been considered as a risk factor in various health outcomes during pregnancy, such as depression and suicide ideation (Wosu et al., 2015; Zhong et al., 2016; Zhang et al., 2020).

However, the relationship between CA and subjective cognitive impairment during pregnancy has received limited attention compared to that given to depression and suicidal ideation. Subjective decline in cognition is a common problem during pregnancy. The phenomenon has been described as “baby brain” (Davies et al., 2018) or “pregnancy brain” (Brown and Schaffir, 2019). Of the cognitive impairments that follow pregnancy, subjective memory impairment is the most frequently cited change (Henry and Rendell, 2007). In one study, over 80% of women complained of forgetfulness during pregnancy, and 38% of women reported that forgetfulness was the only physical or neuropsychological disorder occurring during their pregnancy (Poser et al., 1986). Pregnant women with memory impairment may have problems with skills, tasks, and activities related to planning, thereby affecting their quality of life (Davies et al., 2018). Thus, it is important to explore the relationship between CA and memory impairment in order to help clinicians identify memory impairment in high-risk groups and conduct targeted interventions.

Prior studies explored the relationship between CA and memory impairment in the general population (Goodman et al., 2010). A review showed an association between CA and volume reduction in the prefrontal cortex (PFC) (Hart and Rubia, 2012). The PFC has been found to be related to working memory (Miller and Cohen, 2001). Thus, PFC abnormalities following CA may lead to memory impairment. In addition, CA has been found to be related to subjective memory in adolescents *via* neuroticism (Lin et al., 2017). However, no study has explored the association between CA and memory impairment during pregnancy. During pregnancy, early adverse experiences may reappear during this transition to motherhood, activating the cognitive and emotional responses associated with those childhood experiences (Stockl and Gardner, 2013). These responses may exacerbate memory decline.

Thus, the first aim of this study was to explore the relationship between CA and subjective memory impairment during pregnancy. We focused on subjective prospective memory and retrospective memory based on the following considerations. In case study interviews (Brett and Baxendale, 2001), women complained that they required greater reliance on note taking for organizing work and that they frequently forgot appointments. These are examples of prospective impairments that affect memory for events in the future and retrospective impairment that affects memory of past events. The second aim of this study was to explore the independent and joint associations between different types of CA and subjective memory impairment. Because of the high coincidence among different types of CA, the precise relationship with subjective memory impairment is likely not to be determined accurately if the independent effects of each type are not considered. Overall, this study is the first to explore the association of CA, including PA, EA, and SA, with subjective prospective and retrospective memory impairment during pregnancy. We predicted that there was a relationship between CA and subjective prospective and retrospective memory. We did not establish a prior hypothesis regarding the association of different CA types and subjective memory impairment, as no study has explored the roles of different CA types on memory impairment during pregnancy.

## Methods

This cross-sectional study was conducted at a comprehensive hospital in Shandong province, China, and in accordance with the Declaration of Helsinki. It was approved by the ethics committee of School of Nursing of Shandong University (No. 2017-R-103).

### Participants

Pregnant women at 28 weeks or more of gestation were invited to participate in the study when undertaking a routine prenatal checkup. The inclusion criteria were: (1) age, 18 years or older, and (2) an ability to speak and understand Chinese well. Of the 2,000 eligible pregnant women, 1,825 were willing to participate and completed the questionnaire survey.

### Measurements

#### Childhood Abuse

Childhood abuse, including PA, EA, and SA, was assessed using the Childhood Trauma Questionnaire-Short Form (CTQ-SF) (Bernstein et al., 2003). EA refers to a verbal attack on a sense of worth or well-being of a child, or any humiliating, demeaning, or threatening behavior, directed at the child by an older person (e.g., “The family said hurtful things”). PA refers to forms of physical attack by an older person on a child that are dangerous or lead to harm (e.g., “Hit hard enough to require seeing a doctor”). SA refers to sexual contact between a child and an older person, including explicit coercion (e.g., “Was touched sexually”). CA scored at or above the following threshold was considered as indicating the occurrence of that particular type of CA: PA = 8, EA = 10, and SA = 8. The Chinese version of the CTQ has demonstrated good validity and reliability (Fu et al., 2005). The Cronbach’s α of the CA subscales of the CTQ-SF in this study was 0.811.

#### Subjective Memory Impairment

Subjective memory impairment was assessed using a prospective and retrospective memory questionnaire (PRMQ) (Crawford et al., 2003). This is a 16-item self-report scale with eight items, assessing PM and RM. High PRMQ scores represent high subjective memory impairment. The Chinese version of the PRMQ has demonstrated good validity and reliability (Wu, 2013). The Cronbach’s α of the PRMQ in this study was 0.934.

### Covariates

According to previous studies, age, education, and parity are important factors in relation to memory impairment (Economou, 2009; Kvavilashvili et al., 2009; Glynn, 2012), and were considered as part of this study. Age (year), education (junior high school or less/high school or higher), and parity (nulliparity/multiparity) were self-reported by the participants.

### Data Analysis

Pearson correlation analysis was conducted to explore the bivariate association between CA and subjective prospective and retrospective memory. Since there was no cut-off value in the PRMQ, the dependent variable was continuous, and the multivariable linear regression analysis was used to explore the relationship between CA and subjective prospective and retrospective memory after adjusting for covariates. We established five separate models in all: Model 1, CA total scores; Model 2, EA scores; Model 3, PA scores; Model 4, SA scores; Model 5, only EA/only PA/only SA/both PA and SA/both EA and SA/both EA and PA/all three. Model 5 was conducted to explore the independent effects of different types of CA on subjective prospective and retrospective memory. All models were adjusted for covariates, including age, education, and parity. An alpha level of *p* < 0.05, two-tailed, was used to establish statistical significance.

## Results

The average age of the 1,825 participants was 31.14 (standard deviation: 4.43) years. The average gestation period at the time of questionnaire administration was 38 weeks (standard deviation: 2.36). A total of 6.7% of the respondents had a junior high school level of education or lower. Nulliparity was observed in 46% of the participants. Pearson correlation analysis showed that all types of CA were positively related to prospective and retrospective memory impairment (all *p* < 0.05, see Table 1). After adjusting for covariates, Model 1 in Tables 2, 3 shows that pregnant women with higher CA total scores had a higher subjective prospective (*B* = 0.22, SE = 0.04, β = 0.13, *p* < 0.001) and retrospective memory impairment (*B* = 0.22, SE = 0.04, β = 0.14, *p* < 0.001).

**TABLE 1:** The Pearson correlation analysis of childhood abuse and prospective memory and retrospective memory.

Variables	EA	PA	SA	Prospective memory	Retrospective memory
EA	1				
PA	0.46***	1			
SA	0.47***	0.55***	1		
Prospective memory	0.17***	0.05*	0.08**	1	
Retrospective memory	0.18***	0.07**	0.10***	0.88***	1

EA, emotional abuse; PA, physical abuse; SA, sexual abuse, ***p < 0.001, **p < 0.01, *p < 0.05.

**TABLE 2:** The association between childhood abuse and prospective memory.

Variable	*B*	*SE*	β	*P*
**Model 1**				

Age	–0.03	0.04	–0.02	0.393
Education	0.02	0.54	0.001	0.964
Parity	1.15	0.32	0.10	<0.001
Total childhood abuse scores	0.22	0.04	0.13	<0.001

*F* = 11.32, *df* = 4, adj.*R*^2^ = 0.02				

**Model 2**				

Age	–0.03	0.04	–0.02	0.444
Education	0.01	0.53	0.001	0.978
Parity	1.19	0.31	0.11	<0.001
EA scores	0.59	0.08	0.17	<0.001

*F* = 17.51, *df* = 4, adj.*R*^2^ = 0.04				

**Model 3**				

Age	–0.04	0.04	–0.03	0.317
Education	0.22	0.54	0.01	0.684
Parity	1.12	0.32	0.10	<0.001
PA scores	0.20	0.10	0.05	0.051

*F* = 4.55, *df* = 4, adj.*R*^2^ = 0.01				

**Model 4**				

Age	–0.04	0.04	–0.03	0.300
Education	0.17	0.54	0.01	0.758
Parity	1.13	0.32	0.10	<0.001
SA scores	0.36	0.12	0.07	0.002

*F* = 6.01, *df* = 4, adj.*R*^2^ = 0.01				

**Model 5**				

Age	–0.04	0.04	–0.03	0.248
Education	0.15	0.54	0.0	0.785
Parity	1.18	0.32	0.10	<0.001
Only EA	2.53	1.07	0.06	0.019
Only PA	0.30	0.83	0.01	0.718
Only SA	1.15	0.98	0.03	0.240
Both PA and SA	–1.20	1.99	–0.01	0.546
Both EA and SA	3.51	2.82	0.03	0.213
Both EA and PA	4.42	1.78	0.06	0.013
All three	3.23	1.34	0.06	0.016

*F* = 3.64, *df* = 10, adj.*R*^2^ = 0.01				

*EA, emotional abuse; PA, physical abuse; SA, sexual abuse.*

**TABLE 3:** The association between childhood abuse and retrospective memory.

Variable	*B*	*SE*	β	*P*
**Model 1**				

Age	–0.03	0.03	–0.02	0.423
Education	1.23	0.53	0.06	0.020
Parity	0.77	0.31	0.07	0.013
Total childhood abuse scores	0.22	0.04	0.14	<0.001

*F* = 12.35, *df* = 4, adj.*R*^2^ = 0.03				

**Model 2**				

Age	–0.03	0.03	–0.02	0.468
Education	1.23	0.52	0.06	0.018
Parity	0.80	0.31	0.07	0.009
EA scores	0.56	0.08	0.17	<0.001

*F* = 17.51, *df* = 4, adj.*R*^2^ = 0.04				

**Model 3**				

Age	–0.03	0.04	–0.03	0.345
Education	1.41	0.53	0.06	0.008
Parity	0.73	0.31	0.07	0.019
PA scores	0.23	0.10	0.05	0.022

*F* = 5.33, *df* = 4, adj.*R*^2^ = 0.01				

**Model 4**				

Age	–0.03	0.04	–0.03	0.323
Education	1.36	0.53	0.06	0.010
Parity	0.75	0.31	0.07	0.016
SA scores	0.39	0.11	0.08	<0.001

*F* = 7.07, *df* = 4, adj.*R*^2^ = 0.02				

**Model 5**				

Age	–0.04	0.04	–0.03	0.263
Education	1.37	0.53	0.06	0.010
Parity	0.79	0.31	0.07	0.011
Only EA	2.25	1.05	0.05	0.032
Only PA	0.94	0.81	0.03	0.247
Only SA	1.50	0.95	0.04	0.116
Both PA and SA	–0.88	1.95	–0.01	0.652
Both EA and SA	5.85	2.76	0.05	0.034
Both EA and PA	5.15	1.74	0.07	0.003
All three	2.59	1.31	0.05	0.049

*F* = 4.08, *df* = 10, adj.*R*^2^ = 0.02				

*EA, emotional abuse; PA, physical abuse; SA, sexual abuse.*

We also explored the relationships between the different types of CA and subjective prospective and retrospective memory impairment after adjusting for covariates (see Models 2, 3, and 4 in Tables 2, 3). EA, PA, or SA scores were associated with prospective memory impairment (EA: *B* = 0.59, SE = 0.08, β = 0.17, *p* < 0.001; PA: *B* = 0.20, SE = 0.10, β = 0.05, *P* = 0.051; SA: *B* = 0.36, SE = 0.12, β = 0.07, *p* = 0.002) and retrospective memory impairment (EA: *B* = 0.56, SE = 0.08, β = 0.17, *p* < 0.001; PA: *B* = 0.23, SE = 0.10, *b* = 0.05, *P* = 0.022; SA: *B* = 0.39, SE = 0.11, *b* = 0.08, *p* < 0.001).

Furthermore, we explored the independent and joint effects of CA on subjective prospective memory and retrospective memory impairment. Among pregnant women with only EA, only PA, or only SA, women with only EA were found to have high subjective prospective memory (*B* = 2.53, SE = 1.07, β = 0.06, *P* = 0.019) and subjective retrospective memory impairment (*B* = 2.25, SE = 1.05, β = 0.05, *P* = 0.032). Women with both EA and PA (*B* = 4.42, SE = 1.78, β = 0.06, *P* = 0.013) were found to have high-subjective prospective memory. Women with both EA and SA (*B* = 5.85, SE = 2.76, β = 0.05, *P* = 0.034) or both EA and PA (*B* = 5.15, SE = 1.74, β = 0.07, *P* = 0.003) were found to have high-subjective retrospective memory. Women with all three CA types had high-subjective prospective (*B* = 3.23, SE = 1.34, β = 0.06, *P* = 0.016) and retrospective impairment (*B* = 2.59, SE = 1.31, β = 0.05, *P* = 0.049).

## Discussion

To the best of our knowledge, this study is the first to explore the relationship between CA and subjective memory impairment during pregnancy. Women with high CA total scores had high subjective prospective and retrospective memory impairment. We also explored the independent and joint effects of different types of CA and subjective prospective and retrospective memory impairment during pregnancy. Among pregnant women with only EA, only PA, or only SA, women with only EA were found to have high-subjective prospective and retrospective memory impairment. Women with both EA and PA had high-subjective prospective memory. Women with both EA and SA and both EA and PA were found to have high-subjective retrospective memory. Women with all three CA types had high-subjective prospective and retrospective memory impairments.

Women who had experienced CA were found to have higher-subjective prospective and retrospective memory impairment, which is consistent with the findings of prior studies among other groups such as adolescents (Lin et al., 2017). Our study found that CA has a profound effect on memory function in certain key stages of life, such as pregnancy, which adds to the literature in this area. The following are possible mechanisms that may explain the relationship between CA and memory impairment in adulthood. First, CA can cause stress-induced dysregulation of the hypothalamic-pituitary-adrenal (HPA) axis. For example, it has been shown that CA is associated with greater hair cortisol levels during pregnancy (Schreier et al., 2015). In turn, the HPA axis can influence memory in situations of acute and chronic stress (Wolf, 2003). Thus, CA may affect memory function *via* the HPA axis. Second, existing findings suggest that CA negatively affects the PFC, which plays an important role in cognitive function. It has been found that participants with a history of CA show significantly increased activation in the dorsolateral prefrontal cortex (dlPFC), insula, and precuneus, and significantly decreased activation in the ventromedial prefrontal cortex (vmPFC) during psychosocial stress tasks (Zhong et al., 2019). In addition, evidence from neuropsychological and cognitive neuroscience studies has shown frontal lobe activation during prospective task completion in healthy individuals, suggesting a central role of the PFC in prospective memory (Hashimoto et al., 2011). Therefore, CA may cause prospective deterioration through its deleterious effect on the PFC. Third, emerging evidence has shown that childhood adversity is related to changes in gut microbiota composition during pregnancy (Hantsoo et al., 2019). Specifically, women reporting two or more childhood adverse events had greater differential abundance of Prevotella than the participants with few or no childhood adverse events. In turn, Prevotella abundance has been found to be associated with cognitive function (Panee et al., 2018). Future studies are needed to explore the possible biological mechanisms involved in relation to CA and memory impairment.

Importantly, CA has a major impact on various health outcomes during pregnancy. Because pregnancy involves confronting a role transformation, and abuse is often carried out by parents, early traumatic experiences may resurface, thereby activating cognitive and emotional responses related to childhood experiences (van der Kolk, 1994; Stockl and Gardner, 2013). Our study emphasizes that any adverse effects are also likely to involve memory impairment during pregnancy.

When we considered the independent effects of different types of CA among pregnant women with only EA, only PA, or only SA, only those women who had experienced only EA were found to have higher-subjective memory impairment. One study reported that childhood EA was associated with a significant reduction in predominantly left dorsal medial prefrontal cortex (mPFC) gray matter (GM) volume. This mPFC GM volume reduction was not due to concomitant childhood PA and/or SA, because the reductions were also found when childhood EA was experienced in the absence of concurrent childhood PA and/or SA (van Harmelen et al., 2010). The mPFC plays a role in both recent and remote memories (Hugues and Garcia, 2007). In addition, the findings showed the joint effects of EA and other CA types (PA and/or SA), which emphasize the important role of EA in subjective memory impairment during pregnancy. Significantly, given that variables in this study were measured using self-report questionnaires, the participants who had severe forms of abuse may have higher grade of amnesia (even in relation to their abusive experiences), and this could be the reason for not observing significant association with physical and sexual abuse.

This study has some limitations. First, CA was measured using self-report questionnaires, which could have caused recall bias. Second, we assessed only subjective prospective and retrospective memory impairment. Future studies are needed to assess more different aspects of memory loss, using subjective and objective measures, and explore the association between memory impairment and CA. However, data drawn from subjective memory are considered to have better ecological validity than neuropsychological testing (Bender et al., 2006).

## Conclusion

Childhood abuse is an important public concern worldwide. Our study further expands on the literature in this area. We found a relationship between childhood abuse and subjective prospective and retrospective memory impairment among pregnant women, and that emotional abuse plays a crucial role in subjective prospective and retrospective memory impairment during pregnancy. The results of this study suggest that evaluation of maternal childhood abuse during prenatal care, with special focus on emotional abuse, and providing support if necessary may mitigate the adverse effects associated with childhood abuse.

## Data Availability Statement

The original contributions presented in the study are included in the article/supplementary material, further inquiries can be directed to the corresponding author.

## Ethics Statement

The studies involving human participants were reviewed and approved by the Ethics Committee of School of Nursing of Shandong University. The patients/participants provided their written informed consent to participate in this study. Written informed consent was obtained from the individual(s) for the publication of any potentially identifiable images or data included in this article.

## Author Contributions

XZ performed the manuscript preparation. LW, JW, and FM performed data analysis. JS and DC performed the data collection. FC contributed to the design of the study. All the authors have approved the final draft.

## Conflict of Interest

The authors declare that the research was conducted in the absence of any commercial or financial relationships that could be construed as a potential conflict of interest.

## Publisher’s Note

All claims expressed in this article are solely those of the authors and do not necessarily represent those of their affiliated organizations, or those of the publisher, the editors and the reviewers. Any product that may be evaluated in this article, or claim that may be made by its manufacturer, is not guaranteed or endorsed by the publisher.
